# ESIDE: A computationally intelligent method to identify earthworm species (*E. fetida*) from digital images: Application in taxonomy

**DOI:** 10.1371/journal.pone.0255674

**Published:** 2021-09-16

**Authors:** Saiqa Andleeb, Wajid Arshad Abbasi, Rozina Ghulam Mustafa, Ghafoor ul Islam, Anum Naseer, Irsa Shafique, Asma Parween, Bushra Shaheen, Muhamad Shafiq, Muhammad Altaf, Syed Ali Abbas

**Affiliations:** 1 Biotechnology Laboratory, Department of Zoology, King Abdullah Campus, University of Azad Jammu & Kashmir, Muzaffarabad, AJ&K, Pakistan; 2 Computaional Biology and Data Analysis Laboratory, Department of Computer Sciences & Information Technology, King Abdullah Campus, University of Azad Jammu & Kashmir, Muzaffarabad, AJ&K, Pakistan; 3 Environmental Protection Agency (AJK-EPA), Government of Azad Jammu and Kashmir, Muzaffarabad, AJ&K, Pakistan; 4 Department of Forestry Range and Wildlife Management, The Islamia University of Bahawalpur, Bahawalpur, Pakistan; Feroze Gandhi Degree College, INDIA

## Abstract

Earthworms (Crassiclitellata) being ecosystem engineers significantly affect the physical, chemical, and biological properties of the soil by recycling organic material, increasing nutrient availability, and improving soil structure. The efficiency of earthworms in ecology varies along with species. Therefore, the role of taxonomy in earthworm study is significant. The taxonomy of earthworms cannot reliably be established through morphological characteristics because the small and simple body plan of the earthworm does not have anatomical complex and highly specialized structures. Recently, molecular techniques have been adopted to accurately classify the earthworm species but these techniques are time-consuming and costly. To combat this issue, in this study, we propose a machine learning-based earthworm species identification model that uses digital images of earthworms. We performed a stringent performance evaluation not only through 10-fold cross-validation and on an external validation dataset but also in real settings by involving an experienced taxonomist. In all the evaluation settings, our proposed model has given state-of-the-art performance and justified its use to aid earthworm taxonomy studies. We made this model openly accessible through a cloud-based webserver and python code available at https://sites.google.com/view/wajidarshad/software and https://github.com/wajidarshad/ESIDE.

## 1. Background

Earthworms (Crassiclitellata) also known as rainworms are terrestrial invertebrates, habitually found in soil, eating a wide variety of organic matter [1]. Earthworms normally burrow during the day and consume soil and extract nutrients from decomposing organic matter such as leaves and roots [2]. Earthworms vibrantly affect soil health by transporting nutrients and minerals from below to the surface through their waste and their passageways ventilate the ground. Earthworms being ecosystem engineers significantly affect the physical, chemical, and biological properties of the soil by recycling organic material, increasing nutrient availability, and improving soil structure [3].

Earthworms with more than 6000 extant species constitute a highly diverse group of burrowing annelids [4]. The ecology niche and life strategies of earthworms vary from species to species [4]. Moreover, the presence of more than one species in mixed cultures leads to lower reproduction rates and ineffective ecosystem engineering [4]. Many important activities performed by pharmacologists, farmers, taxonomists, foresters, conservation biologists, and technical personnel of environmental agencies such as monitoring endangered species, studying biodiversity, and determining the impact of climate change depend on accurate species identification. Therefore, the role of taxonomy in earthworm study is significant as without a reliable taxonomy most of the ecological studies are irrelevant [5]. Based on feeding habits and soil profile, earthworms have been classified into three main categories: epigeic, anecic, and endogeic. These parameters are not sufficient to classify earthworms properly and therefore, for the vast majority, nothing is known about their biology and ecology [4, 5].

Mostly, the taxonomy of earthworms is established using different morphological characteristics such as prostomium shape, position, segment number and shape of clitellum, spermathecae, and the arrangements of setae [4, 5]. However, taxonomic classification based on these morphological characteristics is difficult in most of the species and requires a high degree of expertise because the small and simple body plan of earthworms does not have anatomical complex and highly specialized structures [6, 7]. Recent molecular-based techniques such as 16S rDNA, 18S rDNA, and COI sequences have been successfully used as an alternative approach for earthworm identification [6, 8, 9]. However, these technologies need a wide database of DNA sequences of earthworms and involve enormous time and budget. Therefore, there is an utmost requirement for a computational approach that can assist studies to identify and correctly establish the taxonomy of different earthworm species.

In this study, we propose a machine learning-based earthworm species identification model that uses digital images of earthworms. Machine learning has successfully been used to classify different animal species in digital images [10, 11]. Currently, as a pilot study, we have only focused on *Eisenia fetida* (tiger worm) because of its wide range of applications in the field of medicine, pharmaceutical, and agriculture and constraints of availability of data in the form of digital images. *E*. *fetida* possessed anticoagulation and fibrinolytic activity [12], act as an antitumor, antioxidant, wound healing, and antibacterial agents [12, 13], best for vermicomposting [14]. Here, we aim to develop a method that uses a digital image of an earthworm and predict whether it is *E*. *fetida* or not. To the best of our knowledge, this is the first attempt to design such a method to identify earthworm species from digital images.

## 2. Methods

In this section, we give the detail of our methodology adopted to design and develop a machine learning-based earthworm species identification system and its evaluation.

### 2.1. Dataset and preprocessing

For this study, we have collected samples of various earthworm species including *E*. *fetida* from different localities of Azad Jammu and Kashmir, Pakistan. After carefully washing, we took digital images of all the collected samples with a high-quality digital camera (Nikon D5300). After getting high-quality images, we have sorted out these images into two categories *E*. *fetida* and others by consulting taxonomy experts in the field. In this way, we have a dataset of 1240 images of *E*. *fetida* and 772 images of other species.

We have cropped and enhanced all the images in our dataset to be used in the proposed machine learning setting. Cropping involves removing the unwanted area of the image to emphasize earthworm only. We cropped images in our dataset by bounding boxes using Adobe Photoshop (version 19). Different image enhancement techniques such as adaptive histogram equalization (AHE) have also been applied to improve the quality and the local contrast of the images [15]. These enhancement techniques have been applied using a python based tool called Scikit-Image (version: 0.17.2) [16].

### 2.2. Proposed methodology

**Fig 1:**
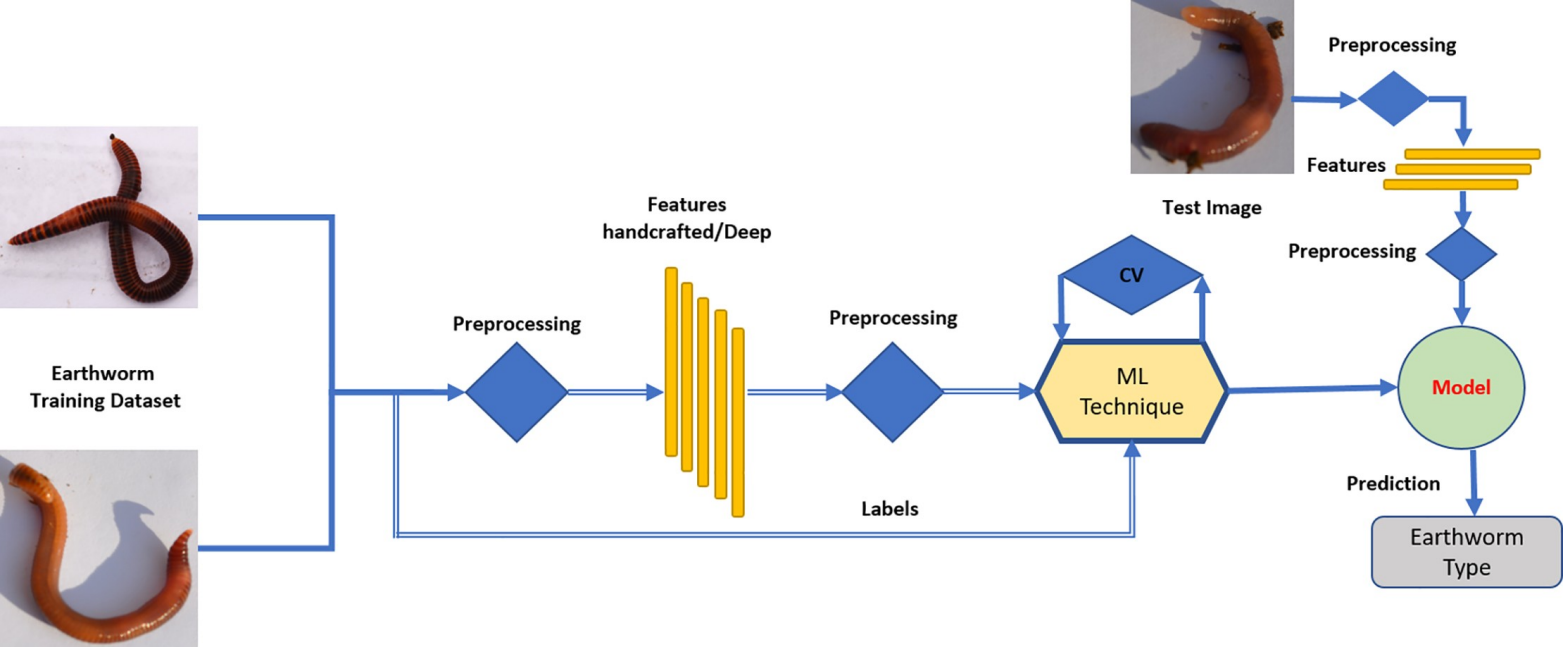
A proposed methodology for the development of computer-aided identification of earthworm species (*E*. *Fetida*) using machine learning and digital images.

We propose a machine learning-based approach for the identification of earthworm species (*E. fetida*) from raw digital images. Various steps involved in earthworm species (*E. fetida*) identification using our proposed scheme are given in Fig 1 and discussed below (please also see S1 Video). We have used conventional (shallow) machine learning models such as support vector machines (SVMs) and transfer learning paradigm instead of deep learning due to data scarcity.

#### 2.2.1. Feature extraction

In image analysis, feature extraction is important as it involves obtaining the most relevant details from the image by reducing dimensionality. If we employ a better feature extraction technique, then it can be expected that the extracted features will better represent the relevant information to perform well over the desired task. In this study, we have used both hand-crafted and deep features extracted using different off-the-shelf CNN based pre-trained models on ImageNet [17]. All of these feature representations *ϕ*(⋅) have been extracted from individual earthworm images. In what follows, we describe the different types of feature representations used in this study.

- **Hand-Crafted Features**

We have used various handcrafted features in this study such as Histogram of Oriented Gradients (HOG) [18], scale-invariant feature transform (SIFT) [19], DAISY [20], Grey Level Co-Occurrence matrix (GLCM) [21], HAAR features [22], Local binary patterns (LBP) [23]. We have extracted these features from all the images in our dataset using Scikit-image (version: 0.17.2) and OpenCV (version: 3.4.2) [16, 24].

- **Deep Feature Maps**

We have used different off-the-shelf CNN-based pre-trained models on ImageNet to extract useful feature maps from the raw digital images of earthworms in our datasets [17]. These pre-trained models include Resnet50 [25], InceptionV3 [26], Xception [27], VGG16 [28], NASNetLarge [29], DenseNet121 [30]. The selection of these pre-trained CNN-based models was based on their reported accuracy. Preprocessing such as pixel scaling and resizing expected by the pre-trained models (varies from model to model) have been applied before extracting the required feature maps. We applied resizing with resampling using pixel area relation through a library for computer vision in python called OpenCV [24].

#### 2.2.2. Classifiers for the identification of earthworm species

In the proposed machine learning setting, we have posed *E. fetida* identification from digital images as a classification problem. For this purpose, we represent each digital image in our dataset as an example of the form (*I_i_, y_i_*) where *I_i_* is an earthworm image and *y_i_* ∈ {+1, −1} is its associated label that indicates whether *I_i_* is *E. fetida* (+1) or not (-1). For a given image *I_i_* in our dataset, we extract hand-crafted features and deep feature maps which can be denoted as a feature vector ***x**_i_*. Our objective is to learn a function *f*(∙) using these feature vectors to identify whether an input image belongs to *E*. *fetida* or some other species. For this purpose, we have used three different classifiers: classical Support Vector Machine (SVM), Random Forest (RF), and Gradient Boosting Machine (XGBoost) [31–33].

- **Support Vector Classification (SVC)**

We have used Support Vector Machines (SVMs) for the detection of earthworm species through a digital image by learning a function *f*(***x***) = 〈***w***,*x*〉 with ***w*** as parameters to be learned from the training data {(***x**_i_, y_i_*)|*i* = 1,2,…,*N*} where, ***x***_*i*_ is the feature representation of an earthworm image *I_i_*. The optimal value of the ***w*** is obtained in SVM by solving the following optimization problem [32].


$$\begin{array}{l} \min_{w,\xi }\frac{1}{2}\lambda \|w{\|}^{2}+\sum_{i=1}^{N}\xi_{i}\\ Such\;that\;for\;all\;i:\;y_{i}\langle w,x_{i}\rangle \geq 1-\xi_{i},\xi_{i}\geq 0 \end{array}$$ (1)

The objective function in Eq (1) maximizes the margin while minimizing margin violations (or slacks ξ) [32]. The hyper-parameter $\lambda =\frac{1}{C}$ controls the tradeoff between margin maximization and margin violation. We used both linear and radial basis function (RBF) kernels and coarsely optimized the values of λ and γ using grid search with Scikit-learn (version: 0.23) [34, 35].

- **Random Forest Classification (RFC)**

Random forest is a supervised learning algorithm that builds an ensemble of decision trees, usually trained with the “bagging” method. A random forest operates by constructing several decision trees in parallel during training and outputs the mean of the classes as the prediction of all trees [31]. It usually performs better on problems having features with non-linear relationships. Each classification tree in the RF is constructed on randomly sampled subsets of input features. In this study, we have optimized RF for the number of decision trees in the forest, the maximum number of features considered for splitting a node, the maximum number of levels in each decision tree, and a minimum number of samples required to split. We have also seen this machine learning technique effectively in use in many other studies [36–39].

- **XGBoost Classification (XGBC)**

XGBoost is a boosting-based ensemble learning technique that chains several weak learners into stronger ones in an iterative way [33, 40]. At the core of XGBoost, there is boosting that lessens biases by supervising the model about what errors have been made by previous models and variance by maneuvering multiple models. In the XGBoost technique, each subsequent model is mentored using the residuals (the variance between the predicted and actual values), then models are fitted via subjective differentiable loss function and gradient descent optimization method by pushing the limits of computational resources for efficient throughput. Here, we used trees as default base learners and optimized XGBoost in terms of the number of boosting iterations, the learning rate, booster, maximum depth, and subsample ratio by employing grid search technique and a python-based package called XGBoost (version: 0.7) [35, 40].

### 2.3. Experimental setup

To train a machine learning-based model and to evaluate its performance to predict the earthworm species from a digital image, we have followed the following experimental setup. We have divided the preprocessed earthworm images into two sub-sets: train-test set (80%), held-out validation set (20%), and reported performance metrics on both the sub-sets. For the train-test set, we have used stratified 10-fold cross-validation (CV). In the stratified 10-fold CV, we have shuffled images in our datasets and then split them into 10 groups by preserving the percentage of samples for each class. 10 models have been trained and evaluated with each group given a chance to be held out as the test set [41]. Average values of performance metrics across folds have been reported in this study. Similarly, to further confirm the robustness of the generalization performance of our proposed technique, we have used the held-out validation dataset to mitigate the possible bias performance improvement under 10-fold CV with hyperparameter tuning using the same training set. For the held-out validation set, we trained the classification models using the whole train-test set and tested them on the validation set. We have used the area under the ROC curve (ROC), the area under the precision-recall curve (PR), and F-measure as performance measures for model evaluation and performance assessment [41–43]. We have computed these metrics using Scikit-learn (version: 0.23) [34]. We used grid search over the training data to find the optimal values of hyper-parameters of different classification models using a python based open-access library for machine learning called Scikit-learn [34, 35]. This automatic grid search was performed once using the train-test set and then the optimum values of hyperparameters have been used during the whole cross-validation process.

### 2.4. Statistical analysis

We have also performed the statistical analysis by checking the statistical significance of obtained performance (F1 score) across different features and classifiers. For this purpose, we have used Wilcoxon test [44]. The test considers the null hypothesis as the median of the performance scores of different models are equal. Alternatively, the performance scores of different models are different. We have used the test statistics at a 95% confidence interval (or α = 0.05). We have performed this analysis using an online webserver (URL: https://tec.citius.usc.es/stac/) [45].

### 2.5. Webserver to identify *E. fetida*

We have developed and deployed a user-friendly cloud-based webserver that uses the optimal machine learning model for *E*. *fetida* identification. This webserver takes an earthworm digital image and predicts whether this image belongs to *E*. *fetida* or not. The webserver is available at https://sites.google.com/view/wajidarshad/software.

## 3. Results and discussion

In this study, we have proposed and developed a machine learning-based computational model to identify earthworm species. For this purpose, we have used a dataset of earthworm digital images, various machine learning algorithms, and different features. In what follows we present results showing the earthworm species identification performance of our proposed method using digital images across different evaluation schemes.

### 3.1. Earthworm species identification performance using handcrafted features

We have trained various classical machine learning models for the classification of *E*. *fetida* versus other earthworm species with a range of handcrafted features and evaluated them using both 10-fold cross-validation (CV) and on an external validation dataset. In both the adopted settings results are shown in Tables 1 and 2. Using 10-fold CV, we observed a maximum F1-score of 0.71 (*p*<0.05) along with 0.75, and 0.86 as the area under the ROC curve, and the area under the PR curve, respectively with Support Vector Classifier and HAAR feature representation (Table 1). The F1 score of 0.71 implies that using a trained machine learning model with SVMs and HAAR features, we have been able to classify *E*. *fetida* correctly approximately 70% of the time. To further confirm the generalization performance of our trained machine learning models with handcrafted features, we have used an external validation dataset. Using an external validation dataset, we observed a maximum F1-score of 0.75 along with 0.77, and 0.85 as the area under the ROC curve, and the area under the PR curve, respectively with Support Vector Classifier and HAAR feature representation (Table 2). We have also observed consistently better performance of HAAR feature representation across RF and XGB classifiers.

**Table 1:** Predictive performance for earthworm species prediction across different classification models and handcrafted features using 10-fold CV (*E. fetida* vs others).

Features	SVC			RFC			XGBC		
	ROC	PR	F1	ROC	PR	F1	ROC	PR	F1
**HOG**	0.69±0.14	0.81±0.09	0.66	0.75±0.13	0.83±0.08	0.64	0.75±0.11	0.85±0.06	0.66
**SIFT**	0.75±0.10	0.84±0.06	0.70	0.74±0.13	0.82±0.11	0.65	0.71±0.10	0.84±0.09	0.68
**DAISY**	0.74±0.12	0.85±0.08	0.71	0.76±0.13	0.86±0.07	0.64	0.75±0.14	0.86±0.08	0.68
**GLCM**	0.69±0.14	0.81±0.09	0.66	0.73±0.13	0.81±0.08	0.63	0.75±0.11	0.84±0.06	0.66
**HAAR**	**0.75±0.15**	**0.86±0.09**	**0.71**	**0.76±0.13**	**0.86±0.07**	**0.65**	**0.75±0.14**	**0.86±0.08**	**0.68**
**LBP**	0.70±0.13	0.80±0.10	0.66	0.65±0.13	0.78±0.10	0.62	0.68±0.12	0.80±0.08	0.65

ROC (Area under the ROC curve), PR (Area under the precision-recall curve), F1 (F1 Score), SVC (Support Vector classifier), RF (Random Forest classifier), XGBC (XGBoost classifier). Bold-faced values indicate the best performance for each model.

**Table 2:** Predictive performance for earthworm species prediction across different classification models and handcrafted features on external validation dataset (*E. fetida* vs others).

Features	SVC			RFC			XGBC		
	ROC	PR	F1	ROC	PR	F1	ROC	PR	F1
**HOG**	0.72	0.82	0.72	0.77	0.81	0.66	0.76	0.84	0.69
**SIFT**	0.77	0.80	0.74	0.77	0.83	0.68	0.73	0.82	0.68
**DAISY**	0.75	0.83	0.73	0.77	0.87	0.68	0.74	0.87	0.70
**GLCM**	0.71	0.80	0.69	0.70	0.80	0.60	0.71	0.83	0.68
**HAAR**	**0.77**	**0.85**	**0.75**	**0.79**	**0.88**	**0.68**	**0.77**	**0.87**	**0.70**
**LBP**	0.72	0.79	0.70	0.68	0.80	0.61	0.68	0.82	0.69

ROC (Area under the ROC curve), PR (Area under the precision-recall curve), F1 (F1 Score), SVC (Support Vector classifier), RF (Random Forest classifier), XGBC (XGBoost classifier). Bold-faced values indicate the best performance for each model.

### 3.2. Earthworm species identification performance using deep feature maps

**Fig 2:**
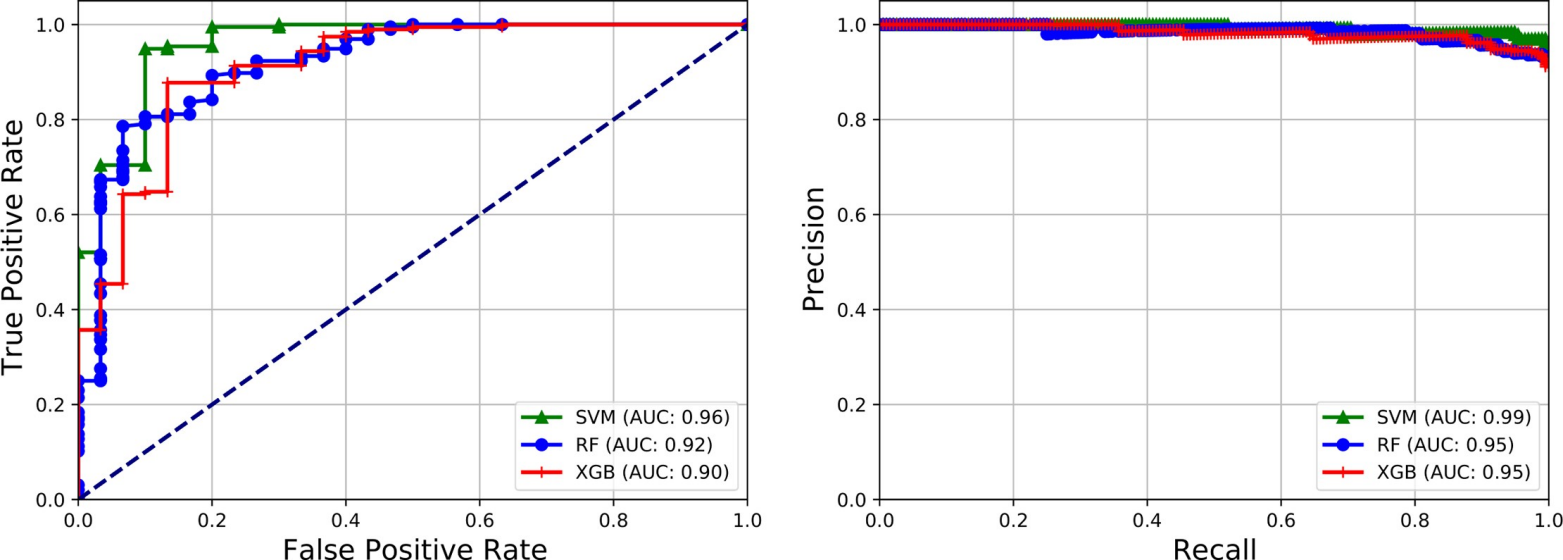
Receiver Operating Characteristic (ROC) and Precision-Recall (PR) curves showing predictive performance of our proposed model for the classification of digital images of earthworms across different classifiers (SVM, RF, XGB) and DenseNet feature map on an external validation dataset. *E. fetida* vs others: ROC(A), PR(B).

We have trained various shallow machine learning models for the classification of *E*. *fetida* versus other earthworm species with a range of deep learning-based feature maps and evaluated using both 10-fold cross-validation (CV) and on an external validation dataset. The results of our evaluation in both settings are shown in Tables 3 and 4 and Fig 2. Using 10-fold CV, we observed a maximum F1-score of 0.80 (*p*<0.05) along with 0.95, and 0.98 as the area under the ROC curve, and the area under the PR curve, respectively with Support Vector Classifier and Densent121 feature map (Table 3). PR score of 0.98 represents high accuracy with fewer false positives (Classifying Other Species as *E. fetida*) and false negatives(Classifying *E. fetida* as Other Species). To confirm further the classification accuracy of our trained machine learning models with deep feature maps, we have used an external validation dataset. Using an external validation dataset, we observed a maximum F1-score of 0.92 along with 0.96, and 0.99 as the area under the ROC curve, and the area under the PR curve, respectively with Support Vector Classifier and Densent121 feature map (Table 4; Fig 2). F1 score of 0.92 and PR score of 0.98 represent a consistently improved performance of our proposed machine learning model to predict *E*. *fetida* class with high precision and recall (i.e. by producing fewer false positives and false negatives). By observing these results obtained through deep feature maps and comparing with the results obtained through handcrafted features, we can easily conclude that deep feature maps perform consistently better across all the classification algorithms. This performance improvement of deep feature maps over handcrafted features has already been reported in a previous study on X-ray scans [46]. These results justify the use of the proposed earthworm species classification model in a real setting.

**Table 3:** Predictive performance for earthworm species prediction across different classification models and deep feature maps using 10-fold CV (*E. fetida* vs others).

Feature Map	SVC			RFC			XGBC		
	ROC	PR	F1	ROC	PR	F1	ROC	PR	F1
**DenseNet121**	**0.95±0.04**	**0.98±0.02**	**0.80**	**0.89±0.07**	**0.95±0.03**	**0.77**	**0.92±0.07**	**0.96±0.03**	**0.80**
**Resnet50**	0.84±0.12	0.88±0.07	0.74	0.78±0.15	0.86±0.10	0.70	0.80±0.15	0.88±0.08	0.70
**Xception**	0.90±0.07	0.95±0.04	0.77	0.85±0.08	0.93±0.05	0.74	0.90±0.07	0.94±0.04	0.75
**InceptionV3**	0.88±0.05	0.90±0.07	0.76	0.80±0.15	0.88±0.08	0.72	0.86±0.13	0.92±0.06	0.76
**VGG16**	0.93±0.05	0.97±0.03	0.77	0.87±0.08	0.93±0.05	0.70	0.90±0.05	0.94±0.03	0.78
**NASNetLarge**	0.82±0.12	0.90±0.07	0.70	0.78±0.13	0.88±0.07	0.70	0.83±0.12	0.90±0.07	0.71

ROC (Area under the ROC curve), PR (Area under the precision-recall curve), F1 (F1 Score), SVC (Support Vector classifier), RF (Random Forest classifier), XGBC (XGBoost classifier). Bold-faced values indicate the best performance for each model.

**Table 4:** Predictive performance for earthworm species prediction across different classification models and deep feature maps on external validation dataset (*E. fetida* vs others).

Feature Map	SVC			RFC			XGBC		
	ROC	PR	F1	ROC	PR	F1	ROC	PR	F1
**DenseNet121**	**0.96**	**0.99**	**0.92**	0.92	0.95	0.85	**0.90**	**0.95**	**0.92**
**Resnet50**	0.90	0.92	0.90	**0.93**	**0.97**	**0.86**	0.88	0.95	0.91
**Xception**	0.88	0.90	0.88	0.86	0.90	0.87	0.87	0.91	0.87
**InceptionV3**	0.92	0.94	0.92	0.84	0.96	0.89	0.88	0.97	0.87
**VGG16**	0.90	0.93	0.92	0.90	0.96	0.88	0.90	0.93	0.89
**NASNetLarge**	0.86	0.90	0.87	0.84	0.88	0.84	0.88	0.97	0.87

ROC (Area under the ROC curve), PR (Area under the precision-recall curve), F1 (F1 Score), SVC (Support Vector classifier), RF (Random Forest classifier), XGBC (XGBoost classifier). Bold-faced values indicate the best performance for each model.

### 3.3. Predictive performance of the proposed model under a real setting

**Fig 3:**
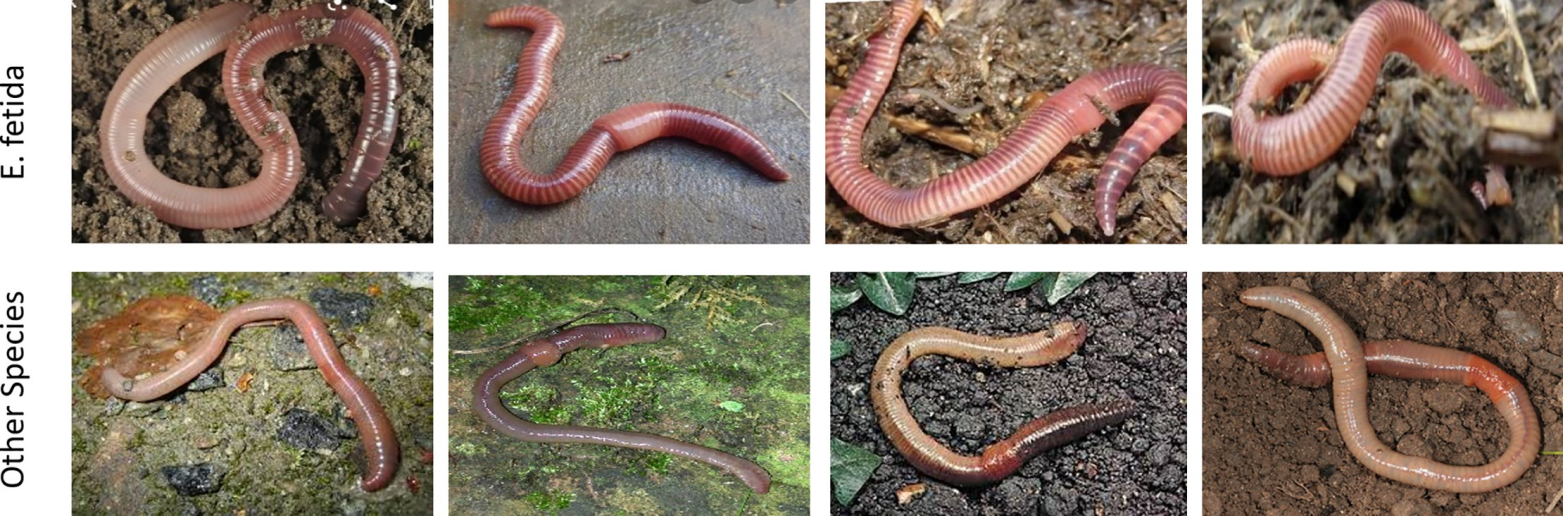
Some of the images of earthworm species (*E. fetida* and other) used to test ESIDE in a real use under the supervision of a qualified taxonomist.

**Fig 4:**
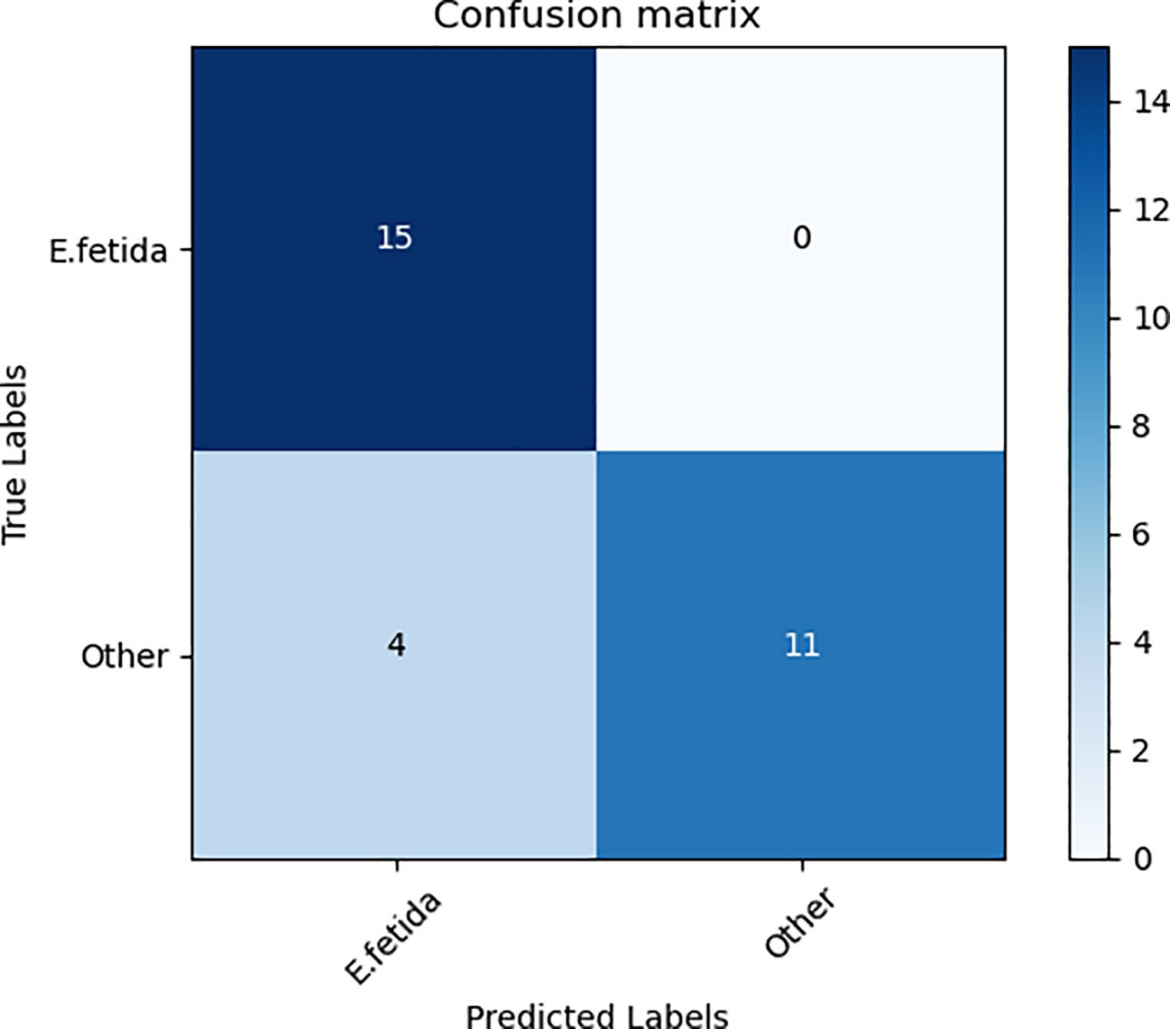
Confusion matrices: Showing the performance of our proposed model for earthworm species identification in a real setting under the supervision of a qualified taxonomist.

We have also checked the generalization performance of our best-trained model for earthworm species identification in a real setting under the supervision of an experienced taxonomist at the Vermi Tech Unit, University of Azad Jammu and Kashmir. For this purpose, we have used 30 digital images of different classes (15 *E*. *fetida*, and 15 other species). A subset of these images is shown in Fig 3. Results obtained through this evaluation are shown as a confusion matrix in Fig 4. Our proposed system (ESIDE) has been able to classify correctly 15 out of 15 provided images of *E. fetida* (Fig 4). Similarly, for the provided images of other species, our system classified correctly 11 out of 15 images, and 4 as *E*, *fetida* (Fig 4). These results show a reasonable performance of our proposed system and justify the use of this model in real settings.

## 4. Conclusions and future work

In this study, we have proposed a machine learning-based model called ESIDE to classify earthworm species by using digital images. We have used both deep feature maps and handcrafted features in this study. Through a series of simulation experiments using both types of features and three different classification algorithms, we have shown that deep feature maps perform consistently better in comparison to handcrafted features while identifying earthworm species through digital images. The stringent performance evaluation through 10-fold CV, on an external validation dataset, and in a use under real settings show that our proposed system can effectively be used to identify *E*. *fetida* from a digital image. The use of our proposed model can aid biologists in taxonomical studies of earthworms. We have made our proposed system accessible through a publically open cloud-based webserver and open-source code. In the future, we will try to develop a generic model for the identification of maximum species of earthworm by incorporating more data.

## Supporting information

S1 Video A short video showing the scientific significance, workflow and design of the current study. (M4V)
