# Supplementary material for: ESIDE: A computationally intelligent method to identify earthworm species (E. fetida) from digital images: Application in taxonomy
Source: PLoS One. 2021 Sep 16;16(9):e0255674. doi: 10.1371/journal.pone.0255674 (PMC8445633; doi:10.1371/journal.pone.0255674)
Supplement: Attachment — Submitted filename: Revision Summary.docx [file pone.0255674.s002.docx]

# Author’s Response to Reviewers' comments

## Summary of the response:

The authors would like to thank the reviewer and the academic editor for their valuable comments on our work. We believe that we have been able to significantly improve the quality of our manuscript based on these comments.

Below is a point-by-point response to these reviewer’s and academic editor’s comments.

## Point-by-Point Responses:

Below we provide a point-by-point response to the comments by the reviewer and outline the actions we have taken in the paper due to these comments. The original comments are given in black whereas our response is given in blue color.

### **Reviewer#1:**

The authors proposed a method for identifying earthworm species (Eisenia fetida) based on machine learning. For that, they evaluated different resource extractors (handcrafted and deep) applied in three classifiers (SVM, RF, and XGBoost). The methotology and written results are convincing and detailed. Recomended the acceptance of the paper with the reservation of itens below:

**Reviewer Comment:**

- High similarity with text "COVIDX: Computer-aided diagnosis of Covid-19 and its severity prediction with raw digital chest X-ray images" available at . On page 6, the phrase "We used SVM for the diagnosis of COVID-19 and its severity prediction by learning a function...", addresses the context and description used in the work "COVIDX". I strongly recommend changing the similar items and adapting to the context of the work.

**Response:**

Thank you for this nice comment and suggestion. We adopted the same methodology to some extent as reported in COVIDX. Now, we have updated this and adapted it to the context of the work.

**Reviewer Comment:**

- I recommend placing the figures and tables in the body of the work and not at the end.

**Response:**

Thank you for this suggestion and we have placed figures and tables in the body of the work.

**Reviewer Comment:**

- It would be interesting to compare the results obtained in table 3 with handcrafted features at the external validation data set.

**Response:**

Thank you for this nice suggestion. We have added and compared the results obtained through handcrafted features on the external validation dataset.

**Reviewer Comment:**

- Congratulations on the work.

**Response:**

Thank you for this encouraging comment.

### **Reviewer#2:**

This paper presents an experimental evaluation of different machine-learning models trained from different features to automatically classify a specific Earthworm species (Eisenia fetida) from images. Overall, the paper is well-written and organized, the problem is clearly motivated, and the chosen methods (feature extractors and classifiers) are adequated. However, there is no computing contribution (e.g., a novel model or neural network, etc). Instead, the paper proposes to explore different combinations of feature extractors and classifiers to classify the target Earthworm species. Although the considered models are adequate for this study, experiments confuse, and some parts sound not technical (I will explain them better below). The author should clarify the authors made their codes and trained models available, but the considered images not (they should mention why).

Here, a summary of all my questions, which are detailed in the following section-by-section review.:

**Reviewer Comment:**

- Are there other methods in the literature that classifies Earthworm species from images?

If they exist, the authors should mention them, compare the proposal models (features + classifier), or explain why they do not consider them in experiments..

**Response:**

To the best of our knowledge, no such study exists so far in the literature and we have mentioned this in the last paragraph of “Introduction”.

**Reviewer Comment:**

- How exactly was image preprocessing performed? Does cropping consider the Earthworms' bodies?

After cropping, are all images resized to the same image resolution (width x height)? Which kind of "enhancement" was performed?..

**Response:**

Thank you for pointing this out. Now, all the detail regarding image preprocessing has been mentioned in “2.1. Dataset and Preprocessing”.

**Reviewer Comment:**

The authors claim: "We used grid search over training data to find the optimal values of hyperparameters of different classification models ..."

How exactly did you do that?

Did you find optimum hyper-parameters 10 times? That is, for each resulting training set from the 10-fold cross-validation (most indicated to not bias the experiments)?

If so, which were the best hyper-parameters for each case and model??..

**Response:**

Thank you for mentioning this important point. Now, all the detail regarding hyperparameter tuning has been mentioned in “2.3.Experimental Setup” as follows.

“We used grid search over the training data to find the optimal values of hyper-parameters of different classification models using a python-based open-access library for Machine Learning: Scikit-learn [31,40]. This automatic grid search was performed once using the train-test set and then the optimum values of hyperparameters have been used during the whole cross-validation process.”

**Reviewer Comment:**

If you (correctly) performed 10 grid-searching, which hyperparameters did you consider when training the models with the whole train-test set to evaluate the validation set?

**Response:**

This automatic grid search was performed once using the train-test set and then the optimum values of hyperparameters have been used during the whole cross-validation process. Also, the same process was performed for the validation set.

**Reviewer Comment:**

Why did you decide to separate a "validation set" from the remaining data?

**Response:**

Thank you for the comment. We have mentioned this in “2.3.Experimental Setup” as follows.

“Similarly, to further confirm the robustness of the generalization performance of our proposed technique, we have used the held-out validation dataset to mitigate the possible bias performance improvement under 10-fold CV with hyperparameter tuning using the same training set.”

**Reviewer Comment:**

Authors should define what a shallow machine learning model is.

I understand a "deep machine learning model" is a model trained with a deep neural network (many layers and weights, consequently a huge feature vector).

How about a shallow machine learning model?

**Response:**

Shallow machine learning models mean conventional (Classical) machine learning such as SVMs and random forest. We have mentioned this in the respective sections.

**Reviewer Comment:**

==> TITLE:

Long and with an English mistake: Instead of "Machine learning based", the correct would be: "Machine learning-based" or still "Machine-learning-based." The same mistake happens throughout the manuscript.

**Response:**

The title has been amended.

**Reviewer Comment:**

The importance of the problem is clear. However, despite mentioning some methods (in the literature) that identify Earthworm species from DNA sequences, the author does not mention if there are methods that use images for that.

If they exist, the authors should mention them, compare the proposal models (features + classifier), or explain why they do not consider them in experiments.

**Response:**

Thank you for this important point but to the best of our knowledge, no such method exist so far.

**Reviewer Comment:**

==> Dataset and Preprocessing

Data acquisition is clear and adequate.

It is unclear how the preprocessing was performed. The authors claim: "We have cropped, resized, and enhance all the images in our dataset to be used in the proposed machine learning setting."

Ok, but how was cropping done? How about image resizing?

After looking at figure 1, I have a sense of what the authors did with the images.

Apparently, the authors cropped the images according to the Earthworms' bodies. However, it is unclear if all images were resized to the same resolution/size (I believe so). If so, some images were distorted since each cropped image has a different aspect ratio.

Also, I do not know what kind of "enhancement" the authors have applied to the cropped-and-resized images..

**Response:**

Thank you for these important points. The detail has been added in the respective section.

**Reviewer Comment:**

==> Deep Feature Maps

The same problem here: "Preprocessing and resizing required by the pre-trained models have been applied before extracting the required feature maps.".

**Response:**

This detail has also been added.

**Reviewer Comment:**

==> Model Validation, Selection, and Performance Assessment

The experimental protocol is confusing. Refer to questions #3 -- #5.

**Response:**

This has been rectified accordingly.

**Reviewer Comment:**

==> Results and Discussion

Since the authors did not perform any statistical test, it is inconclusive which model (features + classifier) is the best.

For example, from Table 1, DAISY, SIFT, and HAAR present similar scores (apparently, with no statistical difference - my guess) regardless of the considered classifier.

The same for DenseNet121 and VGG16 in Table 2.

Also, the authors did not report the standard deviations for the F1 score in the 10-fold experiments..

**Response:**

The details of the adopted statistical test have been added in the revised manuscript. Moreover, the standard deviations for the F1 score is not mentioned because it was close to zero.

**Reviewer Comment:**

==> Conclusion

There is no conclusion or comparison between the hand-crafted and deep features. Of course, by analyzing the results (Tables 1 and 2), I can argue deep features are a better and more accurate option than hand-crafted ones. However, there is no this kind of comment neither in the discussion nor in conclusions.

Which method should we choose?

**Response:**

Thank you for pointing out this important point. We have added the required comparison in the results and discussion and also conclude this in the conclusions.

**Reviewer Comment:**

TYPOS AND GRAMMAR MISTAKES

"machine learning based" --> "machine-learning-based"

Use the active voice more;

"(ASIDE)" --> "ESIDE"

**Response:**

Thank you for pointing these typos out and these typos have been rectified in the revised manuscript accordingly..

### **Reviewer#3:**

The authors present a computer vision pipeline to differentiate between earthworm species, a task that heavily relies on specialized personnel. The performed data collection and the implemented solutio are sound. The initiative to make the solution publicly avaialable is also more than welcome. Last, the obtained results seem to be accurate enough to motivate the usage of the proposed solution in real scenearios. In my opinion there are two major issues with the manuscript:

**Reviewer Comment:**

- It lacks proper placement among existing research. Althought the paper is well-motivated and the used tools are referenced, the authors do not compare their proposal against similar works. For instance, are there similar works that use machine learning to classify different species (in cases where specialists are usually required)? Even though your target application (earthworms) might be not have been explored before with such a methodological strategy, related works might provide useful insights and also shed some light into possible limitations. They could also clearly state how novel is your work in comparison with existing solutions.

**Response:**

Thank you for this concern but to the best of our knowledge no such study exist so far in the literature to predict earthworm species from didgtal image intelligently and we have mentioned this in the last paragraph of “Introduction”.

**Reviewer Comment:**

- It lacks proper placement among existing research. Althought the paper is well-motivated and the used tools are referenced, the authors do not compare their proposal against similar works. For instance, are there similar works that use machine learning to classify different species (in cases where specialists are usually required)? Even though your target application (earthworms) might be not have been explored before with such a methodological strategy, related works might provide useful insights and also shed some light into possible limitations. They could also clearly state how novel is your work in comparison with existing solutions.

**Response:**

Thank you for this concern but to the best of our knowledge no such study exists so far in the literature to predict earthworm species from digital image intelligently and we have mentioned this in the last paragraph of “Introduction”. However, I have cited some related studies to identify species of other animals.

**Reviewer Comment:**

- The procedural descriptions are too sucint. The authors do not provide details regarding their experimental setup and some of the choices they made. For instance, how the model tuning was performed? Which enhancements were applied to the images. Although your source code is publicly available to support reproduciliby, the reader should not need to inpect scripts to be able to understand what was done. Besides, suppose one wants to reproduce your research using other technologies. The paper should provide enough information to do so.

**Response:**

Thank you for these important points. We have tried in the revised manuscript to provide all such details in the relevant sections.

**Reviewer Comment:**

- Some minor comments:

- There are typos and misconstructed phrases throughout the manuscript. Please check the attached file, where some of these occurrences are highlighted.

- Some claims made by the authors are incorrect. For example, the RF variant applied by authors is a classification algorithm, not a regression one, as stated in the manuscript.

- In page 6, the authors mention that they used SVM to predict the diagnosis of COVID-19. This sentence seems to be out of place. Perhaps it came from another paper from the same authors..

**Response:**

Thank you for pointing out these typos. We have corrected all these mistakes in the revised version.

### **Reviewer#4:**

The paper proposes a classification setting using off-the-shelf classifiers and feature extractors for the binary classification of earthworms from images. The context and motivation are relevant, but the paper lacks a deeper analysis and discussion. More specifically, my main concerns are related to the experimental evaluation:

**Reviewer Comment:**

- Details of how the images were cropped and resized are dubious. The authors state: "We have cropped, resized, and enhance all the images in our dataset to be used in the proposed machine learning setting." How was this task performed? Did the authors crop the minimum bounding rectangle (MR) of the earthworm?.

**Response:**

Thank you for pointing out these important issues. We have added these details in the respective sections of the revised version.

**Reviewer Comment:**

- How are the images from the dataset used for validation? Did the authors take the photos using homogeneous background? Please, provide a complete description and some examples..

**Response:**

No images in the validation dataset do not have homogenous background. We have added some examples in Fig. 3.

**Reviewer Comment:**

- How do different brightness levels interfere with the classification results? This analysis is relevant considering that specialists can take photos in ambients that are directly influenced by the solar lighting (or the lack of it).

**Response:**

Images in our train, validation, and relay set all have different backgrounds and brightness level. So the evaluation protocol is stringent keeping in view the directly influenced by the solar lighting.

**Reviewer Comment:**

- How does this unbalanced dataset (1,240 positives x 772 negatives) affect the classification results? Maybe the authors could also show the results using a balanced setting of this experiment.

**Response:**

We have tried training and evaluation by sampling randomly 772 positive examples and found minute changes in the results.

**Reviewer Comment:**

- What is the ratio of pos/neg samples in the folds and the corresponding results?

**Response:**

Thank you for this point. We have used 10-fold stratified cross-validation (CV). In a 10-fold stratified CV, we have shuffled images in our datasets and then split them into 10 groups by preserving the percentage of samples for each class. We have updated this information in the respective section of our revised manuscript.

**Reviewer Comment:**

- IMHO, as it is, the results only point to a generic framework applicable to a binary image classification problem.

A deeper discussion of how different classifiers/features perform better than others in the classification tasks should be provided.

For example: does color, texture, or features classifies the species better? Does the feature dimensionality somehow influence the classifiers?

**Response:**

This kind of discussion has been added in the respective sections.

**Reviewer Comment:**

- The lack of numbering in the section headings and subsections makes the paper organization odd and confusing.

For example: is "Feature Extraction" a subsection of "Proposed Methodology"?

**Response:**

Thank you for this concern, we have added numbering in the section headings in the revised MS.
