# Supplementary material for: ESIDE: A computationally intelligent method to identify earthworm species (E. fetida) from digital images: Application in taxonomy
Source: PLoS One. 2021 Sep 16;16(9):e0255674. doi: 10.1371/journal.pone.0255674 (PMC8445633; doi:10.1371/journal.pone.0255674)
Supplement: Attachment — Submitted filename: Revision Summary.docx [file pone.0255674.s003.docx]

# Author’s Response to the academic editor’s and Reviewers' comments

## Summary of the response:

The authors would like to thank the reviewers and the academic editor for their valuable comments on our work. We believe that we have been able to significantly improve the quality of our manuscript based on these comments.

Below is a point-by-point response to these reviewer’s and academic editor’s comments.

## Point-by-Point Responses:

Below we provide a point-by-point response to the comments by the reviewer/ academic editor and outline the actions we have taken in the paper due to these comments. The original comments are given in black whereas our response is given in blue color.

### **Academic editor:**

I would like to congratulate the authors for there innovative research related to earthworm taxonomy using digital images. This paper presents an initial work to classify earthworm species from digital images which is the first study of its kind reported from any where, therefore the paper has the merit for publication in the journal ,however the manuscript would become more worthy of publication if the authors would incorporate some information related to details about the tools which have been used. The authors also need to incorporate the comments put forward by the second reviewer.

**Response:**

Thank you for these encouraging comments. We have tried to incorporate all the information in the revised manuscript related to the tools used in this study.

### **Reviewer#2:**

The title of the manusript is really good and the information in this paper is new and relevant. But, just give the little basic information about the tools which have been used. And in the result and discussion section the 3rd line "In what follows.......is not clear rephrase the sentence.

**Response:**

Thank you for this feeadback. We have tried to incorporate all the information in the revised manuscript related to the tools used in this study. Also, the sentence in the result and discussion section has been rephrased to make it more clear.
