# Supplementary material for: ESIDE: A computationally intelligent method to identify earthworm species (E. fetida) from digital images: Application in taxonomy
Source: PLoS One. 2021 Sep 16;16(9):e0255674. doi: 10.1371/journal.pone.0255674 (PMC8445633; doi:10.1371/journal.pone.0255674)
Supplement: Peer review history

# Author comment: Author response to previous submission

**DOI:** 10.1371/journal.pone.0255674.r001

29 Apr 2021

Attachment Submitted filename: Revision Summary.docx Click here for additional data file.

# Peer review: Decision Letter 0

**DOI:** 10.1371/journal.pone.0255674.r002

**Authors:** Tunira Bhadauria

30 Jun 2021

PONE-D-21-14202

ESIDE: A computationally intelligent method to identify earthworm species (E. fetida) from digital images: Application in taxonomy

PLOS ONE

Dear Dr. Abbasi,

Thank you for submitting your manuscript to PLOS ONE. After careful consideration, we feel that it has merit but does not fully meet PLOS ONE’s publication criteria as it currently stands. Therefore, we invite you to submit a revised version of the manuscript that addresses the points raised during the review process.

Comments

1.I would like to congratulate the authors for there innovative research related to earthworm taxonomy using digital images. 

2.This paper presents an initial work to classify earthworm species from digital images which is the first study of its kind reported from any where, therefore the paper has the merit for publication in the journal. 

3.the manuscript would become more worthy of publication if the authors would incorporate some information related to details about the tools which have been used.

4.The authors also need to incorporate the comments put forward by the second reviewer.

Please submit your revised manuscript by 20 July 2021. If you will need more time than this to complete your revisions, please reply to this message or contact the journal office at plosone@plos.org. When you're ready to submit your revision, log on to https://www.editorialmanager.com/pone/ and select the 'Submissions Needing Revision' folder to locate your manuscript file.

Please include the following items when submitting your revised manuscript:

- A rebuttal letter that responds to each point raised by the academic editor and reviewer(s). You should upload this letter as a separate file labeled 'Response to Reviewers'.
- A marked-up copy of your manuscript that highlights changes made to the original version. You should upload this as a separate file labeled 'Revised Manuscript with Track Changes'.
- An unmarked version of your revised paper without tracked changes. You should upload this as a separate file labeled 'Manuscript'.

If you would like to make changes to your financial disclosure, please include your updated statement in your cover letter. Guidelines for resubmitting your figure files are available below the reviewer comments at the end of this letter.

If applicable, we recommend that you deposit your laboratory protocols in protocols.io to enhance the reproducibility of your results. Protocols.io assigns your protocol its own identifier (DOI) so that it can be cited independently in the future. For instructions see: http://journals.plos.org/plosone/s/submission-guidelines#loc-laboratory-protocols. Additionally, PLOS ONE offers an option for publishing peer-reviewed Lab Protocol articles, which describe protocols hosted on protocols.io. Read more information on sharing protocols at https://plos.org/protocols?utm_medium=editorial-email&utm_source=authorletters&utm_campaign=protocols.

We look forward to receiving your revised manuscript.

Kind regards,

Tunira Bhadauria, Ph.D.

Academic Editor

PLOS ONE

Journal requirements:

When submitting your revision, we need you to address these additional requirements.

1. Please ensure that your manuscript meets PLOS ONE's style requirements, including those for file naming. The PLOS ONE style templates can be found at

https://journals.plos.org/plosone/s/file?id=wjVg/PLOSOne_formatting_sample_main_body.pdf and

https://journals.plos.org/plosone/s/file?id=ba62/PLOSOne_formatting_sample_title_authors_affiliations.pdf

2. We suggest you thoroughly copyedit your manuscript for language usage, spelling, and grammar. If you do not know anyone who can help you do this, you may wish to consider employing a professional scientific editing service. 

Whilst you may use any professional scientific editing service of your choice, PLOS has partnered with both American Journal Experts (AJE) and Editage to provide discounted services to PLOS authors. Both organizations have experience helping authors meet PLOS guidelines and can provide language editing, translation, manuscript formatting, and figure formatting to ensure your manuscript meets our submission guidelines. To take advantage of our partnership with AJE, visit the AJE website (http://learn.aje.com/plos/) for a 15% discount off AJE services. To take advantage of our partnership with Editage, visit the Editage website (www.editage.com) and enter referral code PLOSEDIT for a 15% discount off Editage services.  If the PLOS editorial team finds any language issues in text that either AJE or Editage has edited, the service provider will re-edit the text for free.

Upon resubmission, please provide the following:

● The name of the colleague or the details of the professional service that edited your manuscript

● A copy of your manuscript showing your changes by either highlighting them or using track changes (uploaded as a *supporting information* file)

● A clean copy of the edited manuscript (uploaded as the new *manuscript* file)

3. Thank you for stating the following in the Acknowledgments Section of your manuscript:

“Dr. Saiqa Andleeb acknowledges the support of the Higher Education Commission (HEC) of Pakistan for granting research projects under the National Research Program for Universities (NRPU) and Technology Development Fund (TDF)(Grant ids: NRPU-2907 & TDF-02006).”

We note that you have provided funding information that is not currently declared in your Funding Statement. However, funding information should not appear in the Acknowledgments section or other areas of your manuscript. We will only publish funding information present in the Funding Statement section of the online submission form.

Please remove any funding-related text from the manuscript and let us know how you would like to update your Funding Statement. Currently, your Funding Statement reads as follows:

 “Saiqa Andleeb acknowledges the support of the Higher  the support of the Higher Education Commission (HEC) of Pakistan for granting research projects under the National Research Program for Universities (NRPU) and Technology Development Fund (TDF)(Grant ids: NRPU-2907 & TDF-02006).

The funders had no role in study design, data collection and analysis, decision to publish, or preparation of the manuscript.”

Please include your amended statements within your cover letter; we will change the online submission form on your behalf.

4. We note that you have stated that you will provide repository information for your data at acceptance. Should your manuscript be accepted for publication, we will hold it until you provide the relevant accession numbers or DOIs necessary to access your data. If you wish to make changes to your Data Availability statement, please describe these changes in your cover letter and we will update your Data Availability statement to reflect the information you provide.

Please review your reference list to ensure that it is complete and correct. If you have cited papers that have been retracted, please include the rationale for doing so in the manuscript text, or remove these references and replace them with relevant current references. Any changes to the reference list should be mentioned in the rebuttal letter that accompanies your revised manuscript. If you need to cite a retracted article, indicate the article’s retracted status in the References list and also include a citation and full reference for the retraction notice.

Additional Editor Comments (if provided):

I would like to congratulate the authors for there innovative research related to earthworm taxonomy using digital images. This paper presents an initial work to classify earthworm species from digital images which is the first study of its kind reported from any where, therefore the paper has the merit for publication in the journal ,however the manuscript would become more worthy of publication if the authors would incorporate some information related to details about the tools which have been used. The authors also need to incorporate the comments put forward by the second reviewer.

[Note: HTML markup is below. Please do not edit.]

Reviewers' comments:

Reviewer's Responses to Questions

**Comments to the Author**

1. Is the manuscript technically sound, and do the data support the conclusions?

The manuscript must describe a technically sound piece of scientific research with data that supports the conclusions. Experiments must have been conducted rigorously, with appropriate controls, replication, and sample sizes. The conclusions must be drawn appropriately based on the data presented. 

Reviewer #1: Yes

Reviewer #2: Yes

**********

2. Has the statistical analysis been performed appropriately and rigorously? 

Reviewer #1: Yes

Reviewer #2: Yes

**********

3. Have the authors made all data underlying the findings in their manuscript fully available?

The PLOS Data policy requires authors to make all data underlying the findings described in their manuscript fully available without restriction, with rare exception (please refer to the Data Availability Statement in the manuscript PDF file). The data should be provided as part of the manuscript or its supporting information, or deposited to a public repository. For example, in addition to summary statistics, the data points behind means, medians and variance measures should be available. If there are restrictions on publicly sharing data—e.g. participant privacy or use of data from a third party—those must be specified.

Reviewer #1: Yes

Reviewer #2: Yes

**********

4. Is the manuscript presented in an intelligible fashion and written in standard English?

PLOS ONE does not copyedit accepted manuscripts, so the language in submitted articles must be clear, correct, and unambiguous. Any typographical or grammatical errors should be corrected at revision, so please note any specific errors here.

Reviewer #1: Yes

Reviewer #2: Yes

**********

5. Review Comments to the Author

Please use the space provided to explain your answers to the questions above. You may also include additional comments for the author, including concerns about dual publication, research ethics, or publication ethics. (Please upload your review as an attachment if it exceeds 20,000 characters)

Reviewer #1: This paper presents an initial work to classify earthworm species from digital images.

The work is simple: a comparison between the combination of a bunch of feature extractors and classifiers.

However, this seems to be the first work to be done in this specific application.

The authors have clarified all my points/questions from the previous version/submission.

Reviewer #2: The title of the manusript is really good and the information in this paper is new and relevant. But, just give the little basic information about the tools which have been used. And in the result and discussion section the 3rd line "In what follows.......is not clear rephrase the sentence.

**********

6. PLOS authors have the option to publish the peer review history of their article (what does this mean?). If published, this will include your full peer review and any attached files.

If you choose “no”, your identity will remain anonymous but your review may still be made public.

**Do you want your identity to be public for this peer review?** For information about this choice, including consent withdrawal, please see our Privacy Policy.

Reviewer #1: No

Reviewer #2: No

[NOTE: If reviewer comments were submitted as an attachment file, they will be attached to this email and accessible via the submission site. Please log into your account, locate the manuscript record, and check for the action link "View Attachments". If this link does not appear, there are no attachment files.]

While revising your submission, please upload your figure files to the Preflight Analysis and Conversion Engine (PACE) digital diagnostic tool, https://pacev2.apexcovantage.com/. PACE helps ensure that figures meet PLOS requirements. To use PACE, you must first register as a user. Registration is free. Then, login and navigate to the UPLOAD tab, where you will find detailed instructions on how to use the tool. If you encounter any issues or have any questions when using PACE, please email PLOS at figures@plos.org. Please note that Supporting Information files do not need this step.

# Author comment: Author response to Decision Letter 0

**DOI:** 10.1371/journal.pone.0255674.r003

3 Jul 2021

Academic editor: 

I would like to congratulate the authors for there innovative research related to earthworm taxonomy using digital images. This paper presents an initial work to classify earthworm species from digital images which is the first study of its kind reported from any where, therefore the paper has the merit for publication in the journal ,however the manuscript would become more worthy of publication if the authors would incorporate some information related to details about the tools which have been used. The authors also need to incorporate the comments put forward by the second reviewer.

Response: 

Thank you for these encouraging comments. We have tried to incorporate all the information in the revised manuscript related to the tools used in this study.

Reviewer#2: 

The title of the manusript is really good and the information in this paper is new and relevant. But, just give the little basic information about the tools which have been used. And in the result and discussion section the 3rd line "In what follows.......is not clear rephrase the sentence.

Response: 

Thank you for this feeadback. We have tried to incorporate all the information in the revised manuscript related to the tools used in this study. Also, the sentence in the result and discussion section has been rephrased to make it more clear.

Attachment Submitted filename: Revision Summary.docx Click here for additional data file.

# Editor report: Decision Letter 1

**DOI:** 10.1371/journal.pone.0255674.r004

**Authors:** Tunira Bhadauria

22 Jul 2021

ESIDE: A computationally intelligent method to identify earthworm species (*E. fetida*) from digital images: Application in taxonomy

PONE-D-21-14202R1

Dear Dr. Abbasi

We’re pleased to inform you that your manuscript has been judged scientifically suitable for publication and will be formally accepted for publication once it meets all outstanding technical requirements.

Within one week, you’ll receive an e-mail detailing the required amendments. When these have been addressed, you’ll receive a formal acceptance letter and your manuscript will be scheduled for publication.

An invoice for payment will follow shortly after the formal acceptance. To ensure an efficient process, please log into Editorial Manager at http://www.editorialmanager.com/pone/, click the 'Update My Information' link at the top of the page, and double check that your user information is up-to-date. If you have any billing related questions, please contact our Author Billing department directly at authorbilling@plos.org.

If your institution or institutions have a press office, please notify them about your upcoming paper to help maximize its impact. If they’ll be preparing press materials, please inform our press team as soon as possible -- no later than 48 hours after receiving the formal acceptance. Your manuscript will remain under strict press embargo until 2 pm Eastern Time on the date of publication. For more information, please contact onepress@plos.org.

Kind regards,

Tuneera Bhadauria, Ph.D.

Academic Editor

PLOS ONE

Additional Editor Comments (optional):

I would like to congratulate the authors for revising the manuscript well, incorporating the suggestions and comments put forward by me and the reviewers in the revised manuscript making it worthy of publication in the journal. Accordingly, I recommend that the manuscript be accepted for publication in the journal.

Reviewers' comments:

# Editor report: Acceptance letter

**DOI:** 10.1371/journal.pone.0255674.r005

**Authors:** Tunira Bhadauria

26 Aug 2021

PONE-D-21-14202R1 

ESIDE: A computationally intelligent method to identify earthworm species (E. fetida) from digital images: Application in taxonomy 

Dear Dr. Abbasi:

I'm pleased to inform you that your manuscript has been deemed suitable for publication in PLOS ONE. Congratulations! Your manuscript is now with our production department. 

If your institution or institutions have a press office, please let them know about your upcoming paper now to help maximize its impact. If they'll be preparing press materials, please inform our press team within the next 48 hours. Your manuscript will remain under strict press embargo until 2 pm Eastern Time on the date of publication. For more information please contact onepress@plos.org.

If we can help with anything else, please email us at plosone@plos.org. 

Thank you for submitting your work to PLOS ONE and supporting open access. 

Kind regards, 

PLOS ONE Editorial Office Staff

on behalf of

Dr. Tunira Bhadauria 

Academic Editor

PLOS ONE
